# Locked in and locked out: sequelae of a pandemic for distressed and vulnerable teenagers in Ireland: Post-COVID rise in psychiatry assessments of teenagers presenting to the emergency department out-of-hours at an adult Irish tertiary hospital

**DOI:** 10.1007/s11845-022-03080-0

**Published:** 2022-06-23

**Authors:** Aoibheann McLoughlin, Ahad Abdalla, Jade Gonzalez, Aoife Freyne, Muhammad Asghar, Yolande Ferguson

**Affiliations:** 1 Department of Psychiatry, Tallaght University Hospital, Dublin 24, Ireland; 2 Centre for Inflammation Research, University of Edinburgh, Edinburgh, Scotland; 3 Crumlin Sector, Tallaght University Hospital, Dublin 24, Ireland; 4 Clondalkin Sector, Tallaght University Hospital, Dublin 24, Ireland; 5 National Drug Treatment Centre, Pearse Street, Dublin 2, Ireland; 6 Dublin South Central Mental Health Service & Psychiatry Department, Tallaght University Hospital, Tallaght University Hospital, Dublin 24, Ireland

**Keywords:** Coronavirus Pandemic, Emergency Department, Psychiatry, Self-Harm, Suicidality, Youth Mental Health, Youth Substance Misuse

## Abstract

**Objective:**

The aim of this study is to investigate the impact of the coronavirus pandemic on teenage psychiatry referrals following crisis presentation to the adult emergency department (ED) of an Irish tertiary hospital. In doing so, this study will specifically examine the effect of COVID-19 on self-injurious behaviour, suicidality and substance use among older adolescents (age 16/17 years).

**Methods:**

This is a retrospective descriptive analysis of acute adolescent psychiatry referrals assessed out-of-hours via the adult ED psychiatry service across three consecutive time points (during the months of March, April and May) from pre-pandemic, 2019 (T1); initial pandemic, 2020 (T2); and peak pandemic, 2021 (T3). Data were obtained via the hospital’s ED-specific electronic database, review of original assessment notes and cross-referenced by manually extracting data logged in the on-call register.

**Results:**

Crisis psychiatry assessments of teenagers during on-call hours trebled during the period of this study (*p* < 0.001). Although ED/crisis referrals initially decreased overall at the start of the pandemic, the rate of teenage referrals remained constant, before increasing as restrictions tightened in lockdown. The negative impact of COVID-19 on teenagers’ ability to cope was found to be statistically significant (*p* = 0.001). Changes in rates of self-harming and/or suicidal behaviours were not statistically significant between 2019, 2020 and 2021 (*p* = 0.082). Alcohol misuse occurred in up to one-third of cases across each timeframe and remained virtually constant throughout the pandemic. Drug misuse decreased from onset of COVID-19 (*p* = 0.01).

**Conclusions:**

To our knowledge, this is the first study to specifically examine the impact of COVID-19 on suicidality, self-harming behaviours, substance misuse and on-call ED presentations of teenagers in Ireland. This study demonstrates that coronavirus-related stress is associated with negative mental health sequelae for vulnerable at-risk older adolescents, as evidenced by a rise in ED presentations and on-call referrals since the onset of the pandemic. Presentation of increased numbers of under-18’s for psychiatry assessment at the adult ED/general hospital indicates a deepening chasm between available and aspirational emergency (adolescent-specific) psychiatric care in the community. Mobilising resilience factors and maximising coping skills for at-risk youth will inform tailored intervention and support strategies along with adequate resourcing of services for vulnerable adolescents in the community.

## Introduction

Suicide is one of the leading causes of teenage mortality worldwide [1, 2], with evidence of a doubling of adolescents presenting with suicidal ideation and suicide attempts to the emergency department (ED) in recent years [3]. In tandem with global trends, self-harm among Ireland’s youth population has increased, along with self-injurious behaviour associated with high lethality [4]. In recognition of increased need, young people with mental health difficulties or risk vulnerabilities have been identified as a priority group for targeted support in *Connecting for Life: Ireland’s National Strategy to Reduce Suicide* [5]. Recommendations include enhancing supports for at-risk youth, incorporation of national anti-bullying and school-based wellbeing/mental health awareness programmes, delivery of early intervention and psychological supports at both primary and secondary care levels and the development of a co-ordinated 24-h/7-day service for those in need of specialist mental health services [5]. At present, ED assessment is often a first point of care [6] for teenagers who present with acute mental health needs, self-harming behaviours and suicidality, (particularly when specialist inpatient and outpatient services are unavailable or inaccessible to them) [7]. As such, careful and considered assessment and care-planning is vital at what can often be a first interface with hospital services.

COVID-19 has changed the complexion of the world as we know it, leading to significant mortality and morbidity worldwide, and placing severe pressure on mental health services. Longitudinal evidence concerning the negative impact on adolescent mental health is emerging, with significant increases in anxiety levels and depressive symptoms, along with a marked decrease in life satisfaction post-implementation of COVID-19 governmental restrictions documented [8]. Surveys evaluating pandemic-associated mental health impacts outline the presence of elevated anxiety and depressive symptoms in adolescents, with higher symptom prevalence reported for females [9–11]. Conflict with parents, difficulties with online learning and pandemic-related stress were found to be associated with mental health difficulties in adolescents [8]. For vulnerable teenagers, restrictions imposed by lockdown, lack of access to support services, periods of home confinement, lack of routine, diminished in-person peer socialisation opportunities and reduced schooling/sports/extra-curricular activities have compounded the impact of isolation and loneliness.

What impact has the coronavirus pandemic had (if any) on ED attendances for adolescents presenting with suicidality and self-harming behaviours? Analysis of rates of suicide-screen results in a US Paediatric ED sample for 6 months in 2019 (pre-pandemic) compared to the same timeframe in 2020 (initial pandemic) highlighted higher rates of suicidal ideation and suicide attempts in specific months throughout 2020 [12]. March and July 2020 showed elevated suicidal ideation for teenagers, while February, March, April and July 2020 demonstrated a higher rate of suicide attempts when compared to the same months in 2019 [12]. An Irish acute hospital study (including older adolescents among its sample) which assessed the impact of COVID-19 on emergency presentations from March to May 2020 in comparison to the same timeframe from 2017 to 2019, demonstrated a reduction in self-harm presentations initially, followed by a marked increase through May 2020 [13].

With this in mind, this study sought to establish the volume and nature of acute psychiatry presentations and referrals of teens aged under-18 years via the adult ED of an Irish tertiary hospital during on-call hours, prior to the pandemic in 2019 (T1), during the initial pandemic phase in 2020 (T2), and at the height of the pandemic restrictions in 2021 (T3).

## Methods

This is a retrospective descriptive analysis of acute adolescent psychiatry referrals assessed out-of-hours via the ED/emergency psychiatry service across three consecutive time points (during the months of March, April and May) from before the pandemic in 2019 (T1), during initial pandemic in 2020 (T2) and during peak pandemic (lockdown period) in 2021 (T3). This study was conducted at Tallaght University Hospital, a Model 4 hospital with co-located adult psychiatry admission unit serving an urban, suburban and rural population of approximately 650,000 people [14] across South Dublin and parts of Kildare. Over 83,000 ED attendances were recorded in 2018 [14], a figure which had decreased to 48,686 attendances in 2020 in the context of the coronavirus pandemic [15].

On-call refers to any hours outside of the general working week/core working hours of 9am–5 pm Mondays to Fridays. It was chosen to reflect a time when the majority of acute ED presentations of under-18’s occur due to resource-driven issues influencing availability of crisis age-appropriate services in the community. In this study, on-call assessment and intervention is typically provided by one on-call Psychiatry Trainee, Locum Registrar or GP Trainee on psychiatry rotation during the hours of 5pm to 9am on weekdays and for 12-hour durations (9am–9 pm) across weekends and bank holidays. Supervision is provided by Senior Registrars and Consultants in adult psychiatry during these times.

Data were obtained via the hospital’s ED-specific electronic database, review of original assessment notes and cross-referenced by manually extracting data logged in the on-call register. All data were pseudo-anonymised and stored on password-encrypted hospital servers. Variables analysed included gender, age, presenting complaint, diagnosis, impact of the pandemic, support structures and follow-up plan. Inclusion criteria consisted of any under-18 attender to the ED referred for psychiatry assessment via the ED during on-call hours, and reviewed either at the ED or subsequently on acute medical assessment or general wards following medical stabilisation. Individuals aged 18 years or over were excluded from this study. To test the change in proportions across years, a chi-square goodness-of-fit test was used. A 2 sample proportion test was used to test for significant changes between the three time-points in respect of self-harm presentations, gender, impact of the pandemic, bullying and use of drugs and alcohol. Significance was set at a *p* value of < 0.05. IBM Statistical Package for Social Sciences version 26.0 was used for data analysis.

## Results

### Volume of teenage attenders

**Fig. 1:**
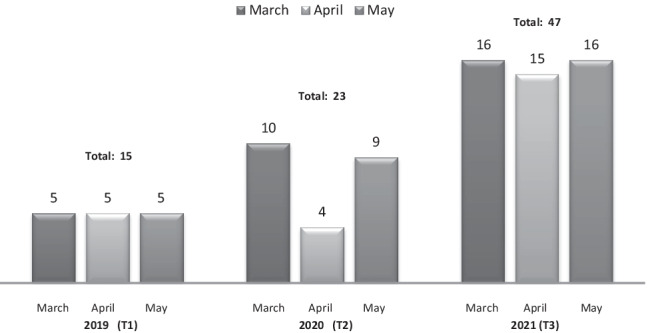
Volume of ED adolescent psychiatry referrals in March/April/May from 2019 to 2021

Crisis psychiatry assessments of teenagers during on-call hours trebled during the period of study evaluation (see Table 1 and Fig. 1). This change in volume was statistically significant (chi-square 19.6 *p* < 0.001).

**Table 1:** Volume of teenagers assessed on-call

2019	Total ED **Ψ** Referrals: 270	Total u-18 **Ψ** Referrals: 15	March: 5 of 97	April: 5 of 81	May:5 of 92	U-18% of Total ED referrals on-call: 5.6%
2020	Total ED **Ψ** Referrals: 234	Total u-18 **Ψ** Referrals: 23	March: 10 of 66	April: 4 of 67	May: 9 of 101	U-18% of Total ED referrals on-call: 9.9%
2021	Total ED **Ψ** Referrals: 334	Total u-18 **Ψ** Referrals: 47	March: 16 of 119	April: 15 of 95	May: 16 of 120	U-18% of Total ED referrals on-call: 14.7%

In 2019, out of a total of 270 on-call ED referrals across the 3-month period of our study, 15 were aged under-18 (March *n* = 5 of 97, April *n* = 5 of 81 and May *n* = 5 of 92). In 2020, there was a reduced overall total of 234 ED on-call referrals across 3 months, 23 of which were under-18 (March *n* = 10 of 66, April *n* = 4 of 67 and May *n* = 9 of 101 referrals). In 2021, out of 334 referrals over a 3-month period, there were a total of 47 under-18 assessments (March *n* = 16 of 119, April *n* = 15 of 95 and May *n* = 16 of 120 referrals). Approximately two thirds of all under-18 referrals in this study presented during on-call hours and were assessed by one on-call clinician. Day-time follow-up and further assessments were also carried out by Liaison Services during core hours.

### Teenagers presenting with self-harm and suicidal ideation

**Fig. 2:**
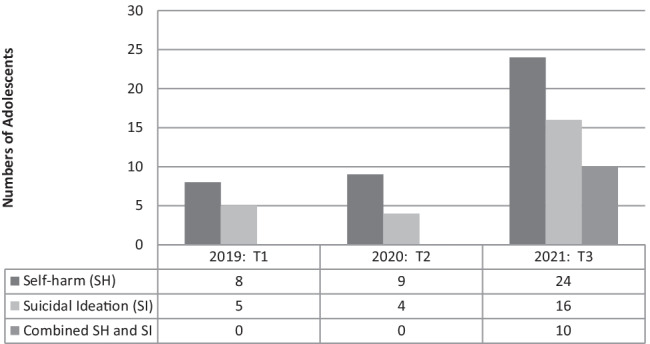
Teenage self-harm and suicidal ideation

Throughout March, April and May 2019, self-harm and suicidality accounted for 13 of the 15 assessments (86.6%) (please see Table 2 and Fig. 2). In the same period in 2020, intentional self-harm and suicidality were present in 13 of the 23 assessments (56.5%). In 2021, self-harm and suicidality occurred as the presenting complaint in 40 of the 47 assessments (85.1%), reverting to pre-pandemic levels. These changes were not statistically significant from 2019, through 2020, to 2021 (*p* = 0.082).

**Table 2:** Self-harm/suicidality and suicidal ideation among teenagers

2019	**Ψ** Referrals (total): 13 of 15	Self-harm: 8	Type of self-harm: -Self-poisoning: 4 -Self-injurious behaviour: 4 (Attempted hanging: 2) (Self-laceration: 2)	Suicidal ideation: 5	Co-morbid self-harm and expressed suicidal ideation: 0
2020	**Ψ** Referrals (total): 13 of 23	Self-harm: 9	Type of self-harm: -Self-poisoning: 5 -Self-injurious behaviour: 4 (Self-lacerations: 4)	Suicidal ideation: 4	Co-morbid self-harm and expressed suicidal ideation: 0
2021	**Ψ** Referrals (total): 40 of 47	Self-harm: 24	Type of self-harm: -Self-poisoning: 13 -Self-injurious behaviour: 11 (Self-laceration: 11)	Suicidal ideation: 16	Co-morbid self-harm and expressed suicidal ideation: 10

### Age and gender

All adolescents presenting to the adult ED in this study were aged 16 (43 of 85) or 17 years (42 of 85), with 55 of overall attenders female (65%) and 30 presenters male (35%). In 2019, females (7 of 15) and males (8 of 15) contributed to roughly half of under-18 presentations. In 2020, females accounted for 14 of 23 presentations (60%) and males presented in 9 of 23 (40%) cases. In 2021, females represented 34 of 47 (72%) attenders, while males comprised 13 of 47 presentations (28%).

In relation to self-harming behaviour and/or suicidal ideation, no significant differences were noted across genders for the months analysed in 2019. In 2020, half of females presented with self-harm/suicidality, while 7 of 9 males presented with self-harm and/or suicidal ideation. In 2021, across the months reviewed, in tandem with rising numbers presenting, more females (10 of 34) attended for treatment of self-poisoning when compared to males (please see Table 3).

**Table 3:** Self-harm/suicidality among teenagers by gender

2019	Total ED **Ψ** Referrals: 15	Total Females: 7	Total Males: 8	Self-harm/suicidality Females: 6/7	Self-harm/suicidality Males: 7/8	Self-poisoning Females: 3/7	Self-poisoning Males: 1/8
2020	Total ED **Ψ** Referrals: 23	Total Females:14	Total Males: 9	Self-harm/suicidality Females: 7/14	Self-harm/suicidality Males: 7/9	Self-poisoning Females: 3/14	Self-poisoning Males: 2/9
2021	Total ED **Ψ** Referrals: 47	Total Females: 34	Total Males: 13	Self-harm/suicidality Females: 29/34	Self-harm/suicidality Males: 10/13	Self-poisoning Females: 10/34	Self-poisoning Males: 2/13

### Impact of COVID-19

**Fig. 3:**
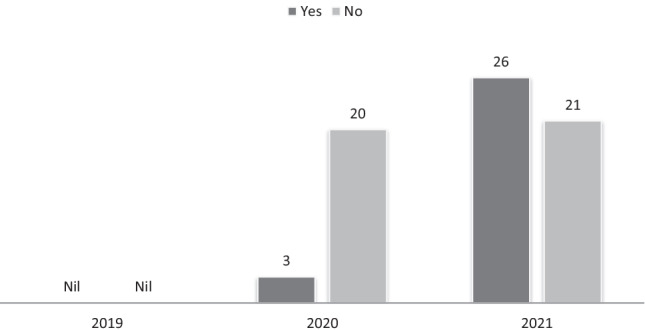
Reported negative impact of COVID-19

Coronavirus-related stress was first documented as a factor influencing crisis presentations in April 2020, with 3 out of the 23 adolescents (13%) highlighting the detrimental impact of COVID-19 in affecting their ability to cope through March, April and May 2020 (please see Fig. 3). This increased to 26 out of 47 adolescents (55%) for the same period in 2021. The impact of COVID-19 was found to be statistically significant (*p* = 0.001).

### Formal supports/follow-up

Child and Adolescent Mental Health Services (CAMHS) were linked in with 59 of the 85 overall number of adolescents (69%) reviewed prior to presentation across 2019, 2020 and 2021. In the pre-pandemic period (T1), 11 of the 15 adolescents (73%) were supported by CAMHS in the community at time of assessment. In 2021 (T3), 30 of the 47 cases (64%) had previous/current CAMHS involvement. Of the overall 85 adolescents assessed, 3 were admitted to Child and Adolescent inpatient units following assessment, and 69 teenagers were referred to outpatient CAMHS (as new or existing referrals) for follow-up intervention in the community.

### Bullying/abuse

This encompasses bullying, abuse or any form of abasement by another person documented as a stressor influencing presentation by the adolescent at time of assessment. In 2019 (T1), 2 of 15 adolescents (13%) reported that bullying/abuse was a factor, while in 2020 (T2), 6 of 23 adolescents (26%) reported that they were affected by abuse or bullying. In 2021 (T3), bullying or abuse was reported by 6 of 47 adolescents (13%). Bullying was not found to be statistically significant (*p* = 0.254) across the years of study.

### Drugs and alcohol

This refers to subjective reports of substance use by adolescent attenders. Alcohol misuse was documented in 5 of 15 cases in 2019 (33%) and 8 of 23 cases (34%) in 2020. In 2021, 15 of the 47 adolescents assessed (31%) reported alcohol misuse. This change was not statistically significant across years (*p* value = 0.971). Drug misuse was endorsed by 8 of 15 (53%) under 18’s in 2019 and 11 of 23 cases (47%) in 2020. In 2021, less cases of drug misuse were evident (9 of 47 cases; 19%). Most frequently used drugs in this study were cannabis, amphetamines, MDMA and benzodiazepines. The decrease in documented drug use from onset of COVID-19 was significant (*p* = 0.01).

## Discussion

To our knowledge, this is the first study to specifically investigate the impact of COVID-19 on suicidality, self-harming behaviours, substance misuse and on-call ED presentations of teenagers in Ireland. Our study demonstrated a threefold increase in adult ED crisis psychiatry referrals of 16- and 17-year-olds in a major Irish tertiary centre since the onset of the pandemic. This is reflected by a trebling of referrals and acute assessments across March, April and May over 3 years from pre-pandemic to peak pandemic. This echoes results demonstrated in an Irish year-long ED study of psychiatric presentations from onset of COVID-19 where the volume of under-18’s referred to psychiatry was noted to have quadrupled [16]. In 2020 (T2), COVID-19 as a trigger/precipitant was reported in 3 of the 23 assessments. In 2021 (T3), pandemic-related factors as a documented source of acute distress had increased to a substantial 26 of the 47 assessments completed. This period coincided with a time of flux where rising coronavirus cases necessitated community restrictions, closure of non-essential services and quarantining remained in place for the majority of this time. At the initial phase of the pandemic (T1) (as knowledge of the virus began to disseminate), social distancing was in a nascent phase, and quarantining entered the nation’s lexicon. At the height of the pandemic in 2021 (T3), lockdown became a reality for Ireland’s teenagers as closure of schools, clubs, sporting activities and face-to-face contact with peers forced a re-configuration of their social, recreational and social spheres.

In tandem with the rise in under-18’s presenting for acute psychiatry review out-of-hours, the percentage of 16- and 17-year-olds presenting with self-harm and suicidal ideation also increased from T2 (2020) to T3 (2021). It is noted that the incidence of self-harm dropped to its lowest level in April 2020, echoing that of a British primary care cohort study which demonstrated a considerable reduction in incidence of self-harm in the same month, prior to returning to pre-pandemic levels by September 2020 [17]. By March, April and May 2021 in this study, teenage suicidality and self-harming rates reverted to pre-pandemic levels, albeit at a higher volume.

Cross-sectional chart reviews of adolescents admitted to psychiatric inpatient care in the USA support a hypothesis that COVID-19 may be contributing towards suicidality in vulnerable, at-risk adolescents as demonstrated by self-reported suicidal ideation and suicide attempt ratings post-pandemic [18]. These adolescents reported that COVID-related suicidal ideation was linked to conflict within the home, financial difficulties, changes in living arrangements and distress at missing out on special or significant events [18].

The majority of teenagers presenting in crisis in this study since onset of the pandemic were of female gender, and were previously linked in with Child and Adolescent Mental Health Services (CAMHS). This correlates with international research indicating that girls are experiencing higher levels of mental health decline than boys in this pandemic-era [8], a phenomenon associated with reduced availability of peer networks (more frequently accessed by girls to buffer significant life stressors), and the increased likelihood of female engagement in help-seeking mechanisms [8, 19]. Results from a worldwide school-based health study across 90 countries on teenagers from varying household income levels demonstrated that, across genders, lack of close and confiding friendships was associated with increased prevalence of suicide attempts in older adolescents aged 16–17 years [20], while feelings of social connectivity have been found to protect adolescents against mental health deterioration during COVID-19 [8]. For already socially isolated teenagers lacking positive or meaningful peer relationships, pandemic restrictions compound this sense of loneliness and offer few opportunities to build up scaffolding of peer support.

Substance misuse infers an increased risk of suicidal ideation and attempts in adolescents [21]. In this study, a minimal reduction in self-reported alcohol misuse from 2019 (T1) to 2021 (T3) was noted. However, a marked reduction in drug misuse was observed from 2019 (53% of attenders) to 2021 (19%). This trend is potentially attributable to community restrictions affecting supply of/access to drugs, cessation of social events and reduced socialisation opportunities within peer groups.

It is suggested that the psychological impact of COVID-19 may pose a higher risk to youth who are socially disadvantaged, living in poverty, experiencing chronic illness, have existing psychopathology, lack opportunities for stress regulation or have experienced maltreatment prior to the pandemic [22]. For vulnerable teenagers living in low-income households, lack of access to resources such as the internet/devices, in addition to lack of space to safely or meaningfully engage online, can adversely affect capacity to engage in educational, recreational and supportive services, while financial pressure, lack of childcare for parents and home-schooling pressures can add to carer burden. For teenagers at risk of abuse and maltreatment, pandemic restrictions may reduce access to preventative child protection and protective statutory/legal services.

According to the HSE CAMHS Operational Guidelines [23], work is ongoing to develop a 7-day out-of-hours service for CAMHS in line with *Connecting for Life (CFL)–Ireland’s National Strategy to Reduce Suicide 2015–2024* [5]. From the data in this study, this is both important and essential in order to relieve pressure on adult mental health emergency services. An international retrospective cohort study spanning ten countries, which evaluated emergency psychiatric assessments of children and adolescents presenting with self-harm during the pandemic (over a 2-month period from 2019 to 2020), also highlighted the need to prioritise intensive community-based outreach services for children and adolescents [24]. In Ireland, current under-resourcing of CAMHS and lack of specialised child-specific crisis community support frequently results in the referral of children aged under-18 to the adult ED for crisis assessment and intervention during on-call hours. In this study, the majority of young people presenting for emergency assessment attended during on-call hours. This correlates with Irish data demonstrating a significant increase in ED presentations (for under-18’s and adults) out of-hours during the COVID-19 pandemic [16]. It is important to highlight that out-of-hours evaluation will primarily be provided by a Trainee in adult mental health who may not be specialised in CAMHS service provision and at a time when access to follow-up supports is not immediately available.

Moreover, conflicting legal frameworks exist concerning physical and mental health care for under-18s. From the age of 16 in Ireland, a person may choose to consent to medical care, and thus will attend an adult ED for this purpose. However, in order for a 16- or 17-year-old to obtain mental health assessment and intervention, consent must first be obtained from the teenager’s parent or legal guardian. If a child requires urgent psychiatric hospitalisation and consent is given, an adult psychiatric ward is wholly inappropriate from a safety and care perspective, and so an inpatient CAMHS bed needs to be sourced. On occasion, it will not be possible to source a CAMHS bed due to resourcing issues. This presents a further care coordination challenge for the on-call clinician. In instances where the acutely ill child or caregiver is not consenting to further psychiatric treatment or hospitalisation, the pathway of care becomes even less clear. The implications here are multivariate, not only in terms of rapid assessment of a vulnerable adolescent presenting in distress during what might be their first point of contact with mental health services, but also considerations of appropriate treatment, placement and forward care-planning. These difficulties are increasingly recognised, and while some progress has been made in terms of CFL, there is room for further development in this regard.

## Limitations

This study addresses crisis ED presentations, self-harm and suicidality in 16- and 17-year-olds (older adolescence) only. This is due to the presence of an on-site Paediatric Emergency Service catering for mental health service provision to teenagers aged 15 and under. As such, information concerning younger adolescents is not captured in this study.

This data-set investigated presentations over a 3-month period duration for 3 consecutive years at one large tertiary hospital site. As such, it offers a snapshot of presentations during this time. More detailed analysis would be offered by analysing adolescent attenders longitudinally for a 3-year period across multiple national sites.

It is likely that the overall volume of on-call cases recorded is an under-estimation of the total volume. This is attributable to verbal referrals which were not recorded on the electronic system or referenced in the on-call register, along with electronic system processing issues.

While the focus of this study centred on out-of-hours attenders/assessments only, inclusion of day-time Liaison assessments would add depth to any further studies.

Substance use data was provided via subjective reporting as routine urine drug toxicology screening was not carried out in all cases. This may represent an under-reporting of overall substance use.

## Conclusions

The advent of coronavirus-related stress, restriction to autonomy, diminished peer networks and social isolation is associated with negative mental health sequelae for vulnerable at-risk older adolescents, as evidenced by a trebling of out-of-hours crisis assessments and negative impact of COVID-19 reported by teenagers across the period of this study. During this time, increased presentations with mental distress mirrored the progression of tightening restrictions and increased morbidity in Ireland as the country struggled to contain rising rates of infectivity.

The temporal association of the pandemic with a rise in crisis presentations of older adolescents to the adult ED sparks a wider debate in terms of specialist community resource and staffing availability in Ireland. This warrants further review, particularly in the context of differing legal parameters around psychiatric care and intervention when compared to medical consent to treatment.

Mobilising resilience factors and maximising coping skills for vulnerable youth will inform tailored intervention and support strategies. Coronavirus implications have deepened the chasm between available and aspirational emergency psychiatric care. Resources will not fully ameliorate the impact of COVID-19 on adolescent mental health, but they would certainly fill what is a considerable and widening gap in the provision of appropriate emergency mental health care.
